# Relationship between cognitive impairment and hand grip strength in a population‐based cohort of older Nigerians: Data from the VALIANT Study

**DOI:** 10.1002/alz.093449

**Published:** 2025-01-09

**Authors:** Rufus O. Akinyemi, Oludotun V Olalusi, Tolulope O Akinyemi, Gabriel O Ogunde, Joseph O Yaria, Eniola O Cadmus, Femi O Popoola, Joshua O. Akinyemi, Mayowa O Owolabi, Roman Romero‐Ortuno, Adesola Ogunniyi, Brian Lawlor

**Affiliations:** ^1^ Neuroscience and Aging Research Unit, Institute of Advanced Medical Research and Training, College of Medicine, University of Ibadan, Ibadan Nigeria; ^2^ University College Hospital, Ibadan, Oyo Nigeria; ^3^ Lead City University, Ibadan, Oyo Nigeria; ^4^ College of Medicine, University of Ibadan, Ibadan, Oyo Nigeria; ^5^ University of Ibadan and University College Hospital, Ibadan, Oyo Nigeria; ^6^ University of Ibadan College of Medicine, Ibadan, Oyo Nigeria; ^7^ Department of Epidemioloy and Medical Statistics, College of Medicine, University of Ibadan, Ibadan Nigeria; ^8^ College of Medicine, University of Ibadan, Ibadan, Oyo State Nigeria; ^9^ Global Brain Health Institute, Trinity College, Dublin Ireland; ^10^ Africa Dementia Consortium, Ibadan, Ibadan Nigeria; ^11^ Global Brain Health Institute, Trinity College Dublin, Dublin Ireland; ^12^ Trinity College Institute of Neuroscience, School of Psychology, Trinity College Dublin, Dublin Ireland; ^13^ St James Hospital, Dublin Ireland

## Abstract

**Background:**

By 2050, the prevalence of dementia is projected to triple with the greatest increases anticipated in Africa and Asia – largely attributable to population growth and cardiometabolic disorder. Hand‐grip strength (HGS) is a known predictor of cardiometabolic and cognitive health. The relationship between HGS and cognitive impairment (CI) among elderly West Africans is not known. We examined the relationship between HGS and cognitive impairment among older adults in a rural community in Ibadan, South West Nigeria.

**Method:**

The Vascular heAlth, fraiLty and cognition in Ageing Nigerians (VALIANT) Study is an ongoing longitudinal community‐based cohort study aimed at exploring the association between cardiovascular health, cognition and frailty in Nigeria. One thousand participants have been recruited via a multistage, stratified cluster random sampling method recruited from a rural community in Ibadan and taken through a battery of cardiovascular, cognitive and frailty assessment tools. Data on HGS, obtained using a digital hand dynamometer, was available for 480 men and women aged≥50years. The Relationship between cognitive impairment (using MoCA <18) and HGS was examined using a multivariable adjusted logistic regression analysis. All associations were reported as ORs with the corresponding 95% confidence intervals (CI).

**Result:**

The mean age of study participants was 64.5 (11.8) with predominant females (65%). Most participants (62.2%) were hypertensivewhile 22.9% were obese. The mean MOCA score in males and females were 22.155.7 and 17.82 6.6 respectively. Approximately 36% had moderate‐severe CI (MoCA<18), 43% had mild CI (MoCA 18‐25), 21% were normal (>25). There was a significant positive correlation between HGS and MoCA 0.45 (p <0.001). The independent determinants of moderate‐severe cognitive impairment in descending order of aOR (95%CI) were age 1.07 (1.03‐ 1.11), higher HGS 0.94 (0.90 ‐0.98), attainment of primary education 0.14 (0.07 – 0.29), secondary education 0.06 (0.02 – 0.16) and tertiary education 0.01 (0.01 – 0.14)

**Conclusion:**

Besides older age and attainment of any form of education, higher HGS was independently associated with moderate‐severe cognitive impairment among elderly Nigerians. Further exploration of the link between HGS and early clinical/neuroimaging biomarkers of dementia is desirable.

